# Health System Barriers for HPV-vaccine uptake in females with a Turkish or Moroccan Migration background in the Netherlands

**DOI:** 10.1093/eurpub/ckac129.184

**Published:** 2022-10-25

**Authors:** J De Zeeuw, J De Zeeuw, JPM Vervoort, DEMC Jansen

**Affiliations:** 1 Department of General Practice and Elderly Care Medicine, University Medical Center Groningen, Groningen, Netherlands; 2 Department of Health Sciences, University Medical Center Groningen, Groningen, Netherlands

## Abstract

**Background:**

HPV vaccine uptake in female adolescents with a Turkish or Moroccan migration background in the Netherlands is lower as compared to the national average. This study aimed to study health system barriers for HPV vaccine uptake in these groups.

**Methods:**

Semi-structured interviews with Turkish/Moroccan (2nd and 3rd degree migrants) female adolescents (12-18 years old), their parents and grandparents were conducted. In addition a focus group discussion with healthcare providers with particular experience with HPV immunization and the target groups was organized. Data was collected between November 2021-April 2022.

**Results:**

22 interviews were conducted among twenty-three individual participants. Sixteen participants with a Turkish migration background were recruited and seven participants were having a Moroccan migration background. In thirteen cases young women in the families did not receive an HPV vaccination. Health system barriers identified were: language barriers in the information provided, focus on HPV being a sexually transmitted disease in information campaigns, lack of knowledge and awareness on HPV among participants and healthcare providers and inability to reach the target groups with tailored immunization programs. Similar health system barriers were mentioned by the healthcare providers, but also included a lack of trust in the government and healthcare institutions, insufficient coordination and collaboration between different healthcare providers. National immunization programs with mass campaign vaccination is mentioned as a barrier to reach families with a Turkish/Moroccan migration background.

**Conclusions:**

Various health system barriers were addressed to be related to HPV vaccine uptake in females with a Turkish/Moroccan migration background in the Netherlands. Despite similarities concerning health system barriers among community members and healthcare providers, different health system barriers were also mentioned.

